# Psychological impact and stress factors among Ha'il medical students post COVID19 pandemic

**DOI:** 10.6026/97320630018392

**Published:** 2022-04-30

**Authors:** Sukaina Kamal Alzedany, Fatima Ismail Alessa, Reem Fahad Alswedani, Ebtehaj Saud Almughais, Fatmah Fahad Alreshidi, Abrar Humoud Fahad Al Lafi, Mashael Alruwayshid, Abeer Suliman Alshuniefi, Sadaf Anwar, Halima Mustafa Elagib

**Affiliations:** 1 College of Medicine, University of Ha'il, Saudi Arabia; 2 Department of Family and community medicine, College of Medicine, University of Ha'il, Saudi Arabia; 3 Department of Family and community medicine, security forces hospital, Riyadh, Saudi Arabia; 4 Department of Family and community medicine,Ministry of health, Riyadh, Saudi Arabia; 5 Department of Biochemistry, College of Medicine, University of Ha'il, Saudi Arabia; 6 Department of Pharmacology, College of Medicine, University of Ha'il, Saudi Arabia; 7 Department of Pharmacology, College of Pharmacy, Omdurman Islamic University, Sudan

**Keywords:** COVID-19, stress, Psychological, Physical, Impact, medical education, mental health

## Abstract

The current COVID-19 pandemic frightfully threatened the whole world, and people in different countries were demanded to be quarantined due to possible contact with the infection. High mortality rate, the spread of COVID19 and the propagation of fake news in social media programs created fear and anxiety among majority of society especially, medical students. One of the most essential changes during the covid-19 was the termination of teaching lectures in physical presence and its replacement by virtual online lectures. Circumstances like these have negative impact on the mental health of medical students. Therefore, it is of interest to investigate the impact of the COVID19 pandemic on medical students’ learning and the effect of distressing situation they experienced, psychological and educational variables specifically during return to physical attendance in college and the impact of these variables of probably affecting factors like age, gender, marital status, transition of preclinical years to clinical years. A Cross-sectional study was completed among medical students at UOH, KSA. The data are collected by distributing an online questionnaire. Statistical analysis has been done with Microsoft Power BI. 14.82% of 5th year female participants were unable to stop worrying for different things while 10.54% of male participant were in the 1st year. Large numbers of students who have increased the number of times they wash their hands are found in med3 and med4 while 27.92% agree and 29.05% strongly agree of whole years of study. 45.29% of students were having mental pressure before online session due to internet connection while 51.55% had decreased motivation since the shift to online learning. Data shows that highest numbers of participants who were having past illness and anxious were in age group 21-23 in both male and female. The majority of students was having fear toward returning in physical presence and preferred not to have on- college education. Hence, it is recommended to encourage students and reduce stress by providing with Personal Protective Equipment (PPE) course.

## Background:

On 31 December 2019 health authorities in Wuhan, china proved that dozens of people had been experienced mysterious pneumonia. On 21 January 2020, USA confirmed it first case after first death from COVID19 in china [1,2]. WHO officially announced this is a pandemic in March [1,3,4]. On the 2nd of March 2020 KSA confirmed its first case. Currently, different types of vaccines are available, there is no cure, and the treatment is supportive depends on patient’s symptoms [5]. Various opinions and coping mechanisms showed by medical students with the additional stress that strike their personal, academic, social lives[6,7]. Different strategies applied to improve mental well-being such as spending time outdoors, exercise, video calls, social media programs, and contemplation [8]. Some studies have found higher level of anxiety and stress between preclinical and clinical medical students during the pandemic in many countries [9]. Other showed that there is a positive response towards sustaining education among the pandemic [10]. The latter studies showed that there are students who wanted to continue suitable education despite difficulties they encountered [11]. Also, medical students worry about missing out education because they belief that will affect their future job as physicians [6]. Depression and anxiety not only impact medical student lives in expressions of their academic execution, professional progress, lack of concentration and impaired memory, but also negatively affect patient care with more clinical errors and less empathy towards patients [12,13]. Medical students particularly facing many unprecedented challenges and factors including fear of catching infection, massive spreads transmission, coping to online learning, assessment way changes, sudden cancellation of clinical postings, all of these may be contributed towards increasing susceptibility of medical students to depression and anxiety [14]. Therefore, it is of interest to investigate the impact of the COVID19 pandemic on medical students’ learning and the effect of distressing situation they experienced, psychological and educational variables specifically during return to physical attendance in college and the impact of these variables of probably affecting factors like age, gender, marital status, transition of preclinical years to clinical years.

## Materials and Methods:

A cross sectional study was conducted from November 2021 to April 2022; on medical student and psychological impact of COVID19 on them during return to physical presence at college of medicine, University of Ha'il (UOH), in kingdom of Saudi Arabia. Medical student's from1st to 5th participated in the study. An electronic questionnaire form consisting of 17 questions was designed and distributed electronically as the methods of collecting data. A total 351 out of 381 participants answered the questionnaire.

## Statistical analysis:

All data were collected; analyzed using Microsoft power BI.

## Results:

Demographic characteristics of the study population: A total of 351 participants successfully completed the study. The higher number of respondents were female, with 53 % and 47% were male. In our study, most of the respondents (55.27%) were between 21-23years of age and single. Other demographic characteristics of the surveyed participants are shown in Table 1(see PDF).

## Levels of anxiety among students by using GAD-7 scale: Feeling nervous, anxious or on edge:

Participants in the age group of 21-23 were highest in numbers with 68 participants having past illness and anxious on several days. However, 66 participants who had past illness were not anxious at all. 28 Participants having past illness reported anxiousness on more than half of the days while 32 participants showed anxiousness every day, as shown in Figure 1(see PDF). Figure 1(see PDF) shows 1 non-Saudi female participant however Saudi participants included 165 male and 185 female. Highest number of apprehensions was reported in the age group of 21-23 with 197 participants. 61 Participants in the age group of 21-23 were having apprehension, also annoyed on several days, while 33 participants were annoyed every day, 43 were annoyed on more than half of the days and 57 were not annoyed at all, as shown in Figure 1(see PDF). Total 96 female participants in the age group of 21-23 had past illness, out of them 34 were anxious on several days. However, 20 female participants had past illness and not anxious at all. 20 Female participants had past illness and reported anxiousness on more than half of the days while 22 female participants showed anxiousness every day, as shown in Figure 2(see PDF). 25 Female participants were having apprehension and were annoyed on several days in the age group of 21-23. 17 Female participants in the age group of 21-23 were having apprehension and annoyed on several days, while only 10 female participants were annoyed on several days in the age group of 18-20, as shown in Figure 2(see PDF).

## Worrying too much about different things and not being able to stop or control worrying:

Total 185 female participants participated in the study, out of them 46 were single and 6 married who worried for different things and were studying in 5th year, as shown in Figure 3(see PDF). Most of the female participants were not at all restless as shown in Figure 3(see PDF). Total 165 male participants participated in the study, out of them 37 male participants who worried for different things were single and studying in 1st year, as shown in Figure 4. Most of the male participants were not at all restless in the first year of their graduation as shown in Figure 4(see PDF).

## Trouble relaxing:

18% out of 50.14% (176 female participants) who were single and studied in fifth year of graduation had highest trouble in relaxing on several days as compared to others. Data shows that single female participants from second year of graduation were the highest in the count for trouble in relaxing nearly every day representing 12% as shown in Figure 5(see PDF).

## Attitudes of medical students towards going to the hospital and having clinic:

### Feeling nervous interacting with colleagues with no masks:

There were 98 male participants in the age group 21-23. Out of these 17 male participants in third year of their education have fear of contracting covid-19. However, they strongly disagreed being nervous while interacting with colleagues with no masks as shown in Figure 6(see PDF).

### I feel anxious going to my clinical rotations and I have increased the numbers of times I wash my hand/ use hand sanitizer while on clinical rotations since the beginning of pandemic:

There were 98 male participants in the age group of 21-23, out of them 16 male participants in third year of their education strongly disagreed to feeling anxious while going for clinical rotations, however, 13 male participants strongly agreed to increased frequency of hand washing as shown in Figure 7(see PDF).

## Attitudes of medical students towards on-collage classes and online learning:

### Feeling more stressed while doing online exam than while on-college exams before the pandemic:

There are 98 male participants in the age group of 21-23 having past illness who study in 3rd, 4th and 2nd year of graduation in increasing order of their count of stress during online exams. 12 Male participants who studied in 2nd year of graduation agreed, 10 disagreed, 1 did not know, 1 strongly agreed, and 5 strongly disagreed to having stress during online exam. However, male participants of the same age group who studied in 3rd year of graduation disagreed mostly; 13 male participants strongly disagreed, 8 strongly agreed, 4 did not know, 7 disagreed, and 3 agreed for having stress during online exams as shown in Figure 8(see PDF).

### Motivation to study has decreased since the shift to online learning:

Out of 98 who studied in 3rd year of graduation, 11 male participants agreed, 4 disagreed, 4 did not know, 9 strongly agreed, and strongly disagreed to decrease in motivation during online learning as shown in Figure 8(see PDF).

## Willingness to return to on-college education:

Medical students of all classes were asked to rate their willingness to return to on-college education on a scale from 1 to 5. 27.63% of the student chose scale one which indicate complete unwillingness to return to on-college education. 27.63% of students are intermediately willing to return to physical presence on scale 3. Data shows that 17.94% of students are completely willing to return to on-college education on scale 5.

## Discussion:

Medical college is known as anxious and stressful environment due to its curriculums and the overload of exams, information and assignments that usually has a negative impact on students' academic performance, psychosocial health, and physical wellbeing [15]. So, COVID-19 has increased this stress by increasing the level of anxiety and fear of contracting the infection. This study aimed at studying the psychological impact of the COVID-19 pandemic on medical students at the University of Ha'il, using a survey sent out to all medical students at UOH to assess their stress. Specifically, during return back to physical presence in college. The majority of respondents in our study were female (53 %) while 47% male participated in the study.55.27% of the respondents were between 21-23years of age and single and this was in a similar finding with a study conducted at American university of Beirut which have almost equally male and female were participated in the study (46.5% and 53.5% respectively) and within the age from 20-24 [16].

Due to Generalized Anxiety Disorder scale (GAD-7 scale) we found that 30.76% of medical students get easily irritated or annoyed on several days, 24.21% on more than half of the days, and 14.81% nearly every day, which proved increased anxiety among them, and this was in harmony with the study in Bangladesh concluded that students who become more agitated currently and those who feel irritated while contacting to human experience increased anxiety and depression symptoms. This agitation might have been due to the disruption of the study, newer study methods, academic activities delay, quarantine at home, and absence of interpersonal communications with peers and partners [17].

Most of the students in our study confirmed that they don't feel anxious while communicating with patients during clinical rotation (31.33% and 27.63% disagreed and strongly disagreed respectively) while (12.25%and 5.12% of students agreed and strongly agreed respectively) that they do feel anxious and this was in agreement with a study done in Beirut(4) which showed that the majority of students reported that they don't feel anxious when interacting with patients during their clinical rotations (47.8% of Med 3 and 46.9% of Med 4 respondents, disagreed or strongly disagreed)while most of the Med 3 students reported that they feel nervous during their rotations (45.5% agreed or strongly agreed). In addition, we found in this study a higher level of anxiety in clinical years students due to transition from preclinical to clinical years and from student to half doctor which is in contradiction to a study conducted in Portugal and found that anxiety was higher in the first year among their sample and decreased in the fourth and fifth years of training [18].

Regarding, high percentage of students who were overwhelmed by online exam (18.23% agreed and 24.50% strongly agreed), assignment and assessments (18.80%agreed and 20.79% strongly agreed), also increased mental pressure before online session due to internet connection (24.78% agreed and 20.51% strongly agreed), and this was the same finding of a study conducted by the College of Wisconsin, USA, which proved that the sudden shift to online education can participated in worsening of already existing mental health problems, this is because of the omission of direct peer interaction, reduced concentration, and the difficulties that some students confront when adapting to the new normal [19].

Most of medical students experienced stress due to the rapid shift in their education showed in American University of Beirut study, which has a similar finding to our study [6]. Several medical schools have been facing difficulties in teaching preclinical medical education similar to our study because of shortage of IT personnel, attacks of cyber online platforms, and time constraints [20]. Furthermore, a study in Japan demonstrated the concerns about the shift toward online education were identified as factors related to depressionn [21]. A study concluded that 63% of students having lack of interaction [22]. Furthermore, Meo et al. found that students showed a sense of emotional segregation from friends and classmates during the quarantine of COVID-19 [23]. The Spread of Colleague-related Burnout (13% vs 17%) had increased due to transition from physical presence to online virtual classes. This recommended that students during online classes are more vulnerable to fatigue and exhaustion of emotions as an outcome of changes in relationships between colleagues and classmates was identified in a study in Madrid [24, 25]. The motivation is higher among students who had positive interaction with colleagues and resulted in higher academic performance which concluded that students have a great desire to communicate with peers [26] also a study conducted in china proved that the departure of physical presence in college and shifting to online platforms had a negative impact, stress and anxiety on their students and made them experienced a kind of separation from their relationships [27] these studies had similar finding as ours, whereas 21.36% and 30.19% agreed and strongly agreed about decreased motivation since the shift to online learning may be due to lack of interpersonal relationship between colleagues and reduced active communication between students and instructors.

Our analyses revealed that 27.63% participants were not interested to return to college owing to the benefits of online classes that resulted in reduced expense of transport, college accommodation rent, and additional meals in college campus. However, our survey finding revealed that 17.94% participant's interest in returning to college for attending off-line classes; the generated graph analysis describes that most of the participants in the age group of 21-23 wanted to return to college, may be due to their previous experience of college life and practical skills that were missing in the online system of classes. Additionally, the participants might be willing to return to college to avoid poor internet connection issues, lack of electronic devices (computer, laptops, and tablets), and insufficient feedback from instructors. Our study correlates well with a study conducted in Singapore, where third of medical students didn't prefer to return to clinical setting during COVID19 pandemic, and higher proportion of them was in the preclinical years [28]. 17.94% participants in our study are completely willing to return to on-collage education suggesting that returning had less psychological impact on them and this may be due to their confidence about protective measurements such as masks and sensitizers, our country efforts in providing us with Tawakkalna application that shows the state of each person whether the students are immunized, infected with COVID19 or not and their emphasis on social distancing which aligned with results of a similar study conducted among Chinese college students showed low occurrence of psychological disorder in their sample which could be due to confidence in prevention measures previous to the resumption of study [29].

## Conclusion: 

Data shows that a higher number of female responses than male indicating higher level of anxiety and stress among females. The majority of students (27.63%) preferred not to return to physical presence, and this is maybe due to fear of contracting COVID-19 infection and lack of protective measurement. Data also shows that online learning reduced the interaction among colleagues and students that lead to anxiety and apprehension. Thus, we recommend encouraging students to change their negative thoughts about off-line college education. We also recommend providing courses to educate students about personal protective equipment especially to the clinical years students to reduce their fear of interacting with patients.
